# The Use of the Anticoagulant Heparin and Corticosteroid Dexamethasone as Prominent Treatments for COVID-19

**DOI:** 10.3389/fmed.2021.615333

**Published:** 2021-04-23

**Authors:** Heloísa Antoniella Braz-de-Melo, Sara Socorro Faria, Gabriel Pasquarelli-do-Nascimento, Igor de Oliveira Santos, Gary P. Kobinger, Kelly Grace Magalhães

**Affiliations:** ^1^Laboratory of Immunology and Inflammation, Department of Cell Biology, University of Brasilia, Brasilia, Brazil; ^2^Département de Microbiologie-Infectiologie et d'Immunologie, Université Laval, Quebec City, QC, Canada; ^3^Centre de Recherche en Infectiologie du CHU de Québec, Université Laval, Quebec City, QC, Canada

**Keywords:** COVID-19, heparin, dexamethasone, anticoagulant, corticosteroid

## Abstract

COVID-19 is spreading worldwide at disturbing rates, overwhelming global healthcare. Mounting death cases due to disease complications highlight the necessity of describing efficient drug therapy strategies for severe patients. COVID-19 severity associates with hypercoagulation and exacerbated inflammation, both influenced by ACE2 downregulation and cytokine storm occurrence. In this review, we discuss the applicability of the anticoagulant heparin and the anti-inflammatory corticosteroid dexamethasone for managing severe COVID-19 patients. The upregulated inflammation and blood clotting may be mitigated by administrating heparin and its derivatives. Heparin enhances the anticoagulant property of anti-thrombin (AT) and may be useful in conjunction with fibrinolytic drugs for severe COVID-19 patients. Besides, heparin can also modulate immune responses, alleviating TNF-α-mediated inflammation, impairing IL-6 production and secretion, and binding to complement proteins and leukotriene B_4_ (LTB_4_). Moreover, heparin may present anti-SARS-CoV-2 potential once it can impact viral infectivity and alter SARS-CoV-2 Spike protein architecture. Another feasible approach is the administration of the glucocorticoid dexamethasone. Although glucocorticoid's administration for viral infection managing is controversial, there is increasing evidence demonstrating that dexamethasone treatment is capable of drastically diminishing the death rate of patients presenting with Acute Respiratory Distress Syndrome (ARDS) that required invasive mechanical ventilation. Importantly, dexamethasone may be detrimental by impairing viral clearance and inducing hyperglycemia and sodium retention, hence possibly being deleterious for diabetics and hypertensive patients, two major COVID-19 risk groups. Therefore, while heparin's multitarget capacity shows to be strongly beneficial for severe COVID-19 patients, dexamethasone should be carefully administered taking into consideration underlying medical conditions and COVID-19 disease severity. Therefore, we suggest that the multitarget impact of heparin as an anti-viral, antithrombotic and anti-inflammatory drug in the early stage of the COVID-19 could significantly reduce the need for dexamethasone treatment in the initial phase of this disease. If the standard treatment of heparins fails on protecting against severe illness, dexamethasone must be applied as a potent anti-inflammatory shutting-down the uncontrolled and exacerbated inflammation.

## Introduction

Severe acute respiratory syndrome coronavirus 2 (SARS-CoV-2), the etiological agent of coronavirus disease (COVID-19), is rapidly spreading worldwide, being the cause of death of more than two million individuals globally in March of 2021 [World Health Organization (WHO), 2020]. Severe COVID-19 occurs due to complications, mostly affecting the lungs (1), heart (2), and kidney (3), impact more frequently the elderly and patients with certain comorbidities, like heart disease and hypertension, and usually correlates with intensive care unit (ICU) admission, mechanical ventilation requirement, and death (4). The elevated numbers of ill and deceased patients due to COVID-19 place it as a healthcare emergency and highlight the urgency of establishing effective drug therapy strategies for pandemics management.

Severe COVID-19 patients tend to show elevated circulating amounts of the proinflammatory mediators Interleukin-6 (IL-6), IL-8, IL-1β, and Monocyte Chemoattractant Protein-1 (MCP-1) (5). Even though plasma concentrations of IL-6, IL-8, and tumor necrosis factor-α (TNF-α) in severe COVID-19 patients may be lower than subjects affected by septic shock and similar to levels found in other patients in a critical state (6), the inflamed phenotype correlates with poor COVID-19 prognosis and is generated by cytokine storm, a condition characterized by macrophage activation syndrome, lymphopenia and organ mononuclear cell infiltration (7). Besides, SARS-CoV-2 infection leads to endothelial damage (8) and downregulation of its cell entry receptor ACE2 (9), further intensifying inflammatory responses and impacting coagulation processes. In this context, severely ill patients may benefit from anti-inflammatory therapies, including dexamethasone.

The hypercoagulable state characteristic of COVID-19 severity is illustrated by the consistently elevated D-dimer levels (10), disseminated intravascular coagulation (DIC), and subsequent consumption coagulopathy (11) displayed by affected individuals. These alterations explain the increased risk of developing life-threatening complications, such as pulmonary embolism (PE) (12) and myocardial infarction (13), by this group. Thus, administrating the anticoagulant drug heparin may also be useful for improving outcomes in severe cases.

Inflammatory and coagulation processes interplay intimately in many ways, including through Tissue Factor (TF), thrombin-dependent PAR activation, Toll-like Receptors (TLRs), and complement system (14). Elevated levels of COVID-19 proinflammatory markers, such as IL-6 and IL-8, also enhance the risk of thrombosis (15). High levels of IL-6 disrupt the procoagulant-anticoagulant balance by increasing the expression of clotting factors and diminishing the levels of thrombogenesis inhibitors (16). IL-8 also impacts hemostasis, significantly changing fibrin and thrombin amounts, and activating platelets (17). Once the intensity of inflammatory responses and clotting processes influence the prognosis of severe COVID-19 patients, investigating the effectiveness of anti-inflammatory and anticoagulant drugs may be crucial in decreasing morbidity and mortality caused by the COVID-19 pandemics. In this review, we discuss the use of the anticoagulant heparin and the anti-inflammatory dexamethasone as promising tools for the management of severe COVID−19 patients.

## Anticoagulant Heparin

The emerging association between thromboembolic events and COVID-19 raised new insights about the physiopathology of the pandemic disease. Countless therapeutic targets were proposed through the knowledge of unbalanced procoagulant/anticoagulant factors that leads to the impairment of endogenous antithrombotic activity during SARS-CoV-2 infection (18–20). The great hypothesis was regarding the administration of anticoagulant drugs that could restore hemodynamic homeostasis and protect against the observed coagulopathy. Based on that, the study of frequently used anticoagulant drugs, especially heparin, increased worldwide and preliminary data suggest promising performance in clinical medicine.

Heparin is a polysaccharide originally isolated from mammalian animal tissue in 1916 (21). Thereafter the discovery, increasing research about the molecule chemical structure and mechanism of action brought new derivatives that improved the efficacy of antithrombotic activity and decreased side effects associated with the unfractionated heparin (UFH) form (22). The UFH was associated with an increased risk for thrombocytopenia and osteoporosis, causing a greater need for monitoring patients during the therapy (23, 24). Thereby, the development of low-molecular-weight heparin (LMWH) reached new perspectives and is currently the anticoagulant of choice for the treatment and prevention of coagulopathies.

Both UFH and LMWH have the ability of binding to anti-thrombin (AT) glycoprotein, enhancing AT inactivation of potent enzymes in the coagulation pathway, such as Factor Xa and Factor IIa (Thrombin). The heparin dependence of AT to prevent blood clot formation makes the drug an indirect antithrombotic agent and the absence of intrinsic fibrinolytic activity impairs thrombi breakdown when they are already shaped (25, 26). Besides, heparins also present an interesting immune-modulatory activity (27). One of the proposed mechanisms is the inhibition of different inflammatory responses mediated through necrosis factor-α (TNF-α), a potent pro-inflammatory cytokine (28, 29). As a result, the quick and efficient actions of heparin make the drug promising against inflammation, in addition to its anticoagulant properties. Thereby, different reports have been exploring this potential against SARS-CoV-2 infection, in which both inflammatory and coagulation disruption can lead to a complication during the disease progression.

Reports have shown that COVID-19 patients presented increased levels of procoagulation biomarkers and severe immune dysfunction that can lead to disseminated intravascular coagulation (DIC), increased risk for venous thromboembolism (VTE), and organ failure, which reflects in a high hospital mortality rate of 1:31 with a confidence interval (CI) of 95% (30–32). Using an animal model, the increased deposition of collagen, fibrin, and von Willebrand factor was linked to augmented thrombi formation and endothelialits establishment during COVID-19 (33). Moreover, the appearance of neurological alterations caused by cerebral venous thrombosis was also reported (34, 35). In this context, early anticoagulant treatment was applied and it was observed a decrease of D dimer levels, mechanical ventilation urgency, and a decrease of 24.2% in the mortality rate of patients presenting sepsis-induced coagulopathy (30, 32, 36, 37). The use of a high dose of UFH, such as 5,000 U, showed important protection against sepsis-induced VTE, a complication that presents a major impact on a patient's prognosis (32). Besides, ongoing clinical trials have been focusing on inhalational heparin treatment for hospitalized patients with or without mechanical ventilation urgency, especially due to several reports that addressed the protective role of nebulized UFH against intrapulmonary fibrin deposition and lung injury (38, 39). The use of this administration route can directly deliver the UFH to the lung microenvironment, being a potential mechanism to soften local hypercoagulation and hyperinflammation, preventing systemic harmful effects of anticoagulant treatment. The above-mentioned and the main in-progress clinical trials are summarized in Table 1.

**Table 1:** Studies on heparin therapy for COVID-19.

**Author, year, study design, country**	**Number of patients**	**Treatment**	**Patient characteristics**	**Results**
Ranuci et al., 2020; Prospective cohort; Italy	16	Use of an intensive thromboprophylaxis protocol with LMWH, antithrombin and clopidogrel	Comorbidities: 5% - obesity (BMI > 30 kg/m^2^); 20% diabetes; 16% CDV	56.3% with progression toward normal coagulation profile, after increased thromboprophylaxis at day 14
Tang et al., 2020; Retrospective cohort; China	449	Use of UFH or LMWH thromboprophylaxis	Comorbidities: 40% hypertension; 21% diabetes and 9.1% CDV	A 20% reduction in mortality was observed when patients with D-dimer exceeding 3.0 μg/mL and were treated with heparin
Zhang et al., 2020; Retrospective cohort; China	143	LMWH prophylaxis	Age: 63 y; Comorbidities: obesity: 24.9 kg/m^2^ - 35.8%; 39.2% hypertension; 18.2% diabetes	8.8% DVT (all the hospital)
Middeldorp et al., 2020; Retrospective cohort; Netherlands	198	Standard and doubled LMWH prophylaxis	Age: 61 y; BMI: 27 kg/m^2^	GW: PE 6.6%, 13% DVT ICU: PE 15%, 32% DVT
Llitjos et al., 2020; Retrospective cohort; France	26 ICU	31% LMWH prophylactic, 69% therapeutic	Age: 68 y; Comorbities: Hypertension: 85%	
Helms et al., 2020; Multicentric prospective cohort; France	150 ICU	70% LMWH prophylactic, 30% therapeutic	Age: 63 y; Comorbiditie: Diabetes: 22.1%	16.7% PE; 2.6% ATE
Fauvel et al., 2020; Muticentric retrospective cohort; France	1,240 non-ICU	8.4% LMWH prophylatic; 11% UFH prophylatic	Age: 64 y; Comorbidities: 45.4% hypertension; 21.7% diabetes;	8.3% VTE
Lodigiani et al., Retrospective cohort; Italy	327	40.7% LMWH prophylatic; 22.6% UFH prophylatic	Age: 68 y; Comorbidities: 29.8% BMI>30 kg/m^2^; 44.3% hypertension; 18% diabetes	6.4% VTE
Klok et al., 2020; Retrospective cohort; Netherlands	184 ICU	Nadroparin (2,850 IUod^*^ increased in some to 5,700 IUbd)	Age: 64 y; Comorbidity: obesity	31%VTE
Van Haren et al., 2020; Prospective cohort; Australia, UK, Argentina, Brazil, and Egypt	712	Inhaled nebulized UFH (and standard care dose 25,000 IU)	Age: 18 y and older with no immediate requirement for mechanical ventilation	
Van Haren et al., 2020; Prospective cohort; Australia, Ireland, USA, Spain, and the UK	202 ICU	Nebulized UFH (25,000 IU)	Age: 18y and older presenting hypoxemia and acute pulmonary opacity	

*ATE, arterial thromboembolic events; DVT, deep vein thrombosis; ICU, intensive care unit; MWH, low molecular weight heparin; PE, pulmonary embolism; VTE, venous thromboembolism; UEDVT, upper extremity deep vein thrombosis*.

* *5,700 IUod for patients >100 kg*.

It is important to emphasize the importance of indicating LMWH and UFH for both prophylaxis and early treatment, once these drugs are not able to promote fibrinolysis of pre-existing thrombi formed in the tissue. Aware of this fact, the combination of heparin and fibrinolytic drugs could be effective for the treatment of severe cases, once thrombolysis also presents clinical benefits against severe pulmonary embolism (40).

Beyond the expected effects of heparins on preventing clots formation through indirect inhibition of the coagulation pathway, other properties provided by these drugs can be the key for the positive data about their efficacy compared to single-target drugs. The exacerbated inflammation that is also found in severe COVID-19 cases is mediated especially through the uncontrolled production of pro-inflammatory cytokines such as interleukin-6 (IL-6), interleukin-8, monocyte chemoattractant protein-1 (MCP-1), and TNF-α in the cytokine release syndrome (41, 42). Research has been emphasizing how targeting TNF-α is important for a better prognosis against COVID-19-induced cytokine dysfunction, once its blockade can reduce both inflammatory and prothrombotic biomarkers (43). In this context, reports have shown that both LMWH and UFH soften TNF-α-induced inflammatory responses, such as the proper IL-6 and IL-8 production (28, 44, 45). This effect may occur through UFH inhibition of the nuclear transcription factor-κB (NF-κB) binding to the DNA, which is a crucial process for a broad range of cytokines signaling pathways (44). Besides, a non-anticoagulant fraction of enoxaparin was reported as a partial inhibitor of IL-6 release, indicating that the signalization range assigned to this cytokine may be mitigated in the presence of LMWH (46). Indeed, a retrospective review has demonstrated that treating COVID-19 cases with LMWH decreases IL-6 overproduction, thus being an important therapeutic choice to be considered, once inflammation and coagulopathies are closely related (37). Studies have also discussed that disturbances on the pro-inflammatory agents that compound the complement cascade may be modulated during COVID-19 illness (47, 48). It was demonstrated that the complement system plays a role in the SARS-CoV-2-induced endothelial damage in rhesus macaques (33). Thereby, it is important to highlight that UFH and LMWH also bind to complement proteins and reduce the classical cascade activity, thus being instigators of anti-inflammatory responses either through this pathway (49, 50).

Eicosanoid lipid mediators have been identified as important agents during the virus-induced inflammatory process of respiratory airways (51), as observed in Influenza (52) and acute severe respiratory syncytial virus bronchiolitis infection (53). Human bronchial epithelial cells and resident leukocytes of the lung are important sources of leukotriene B4 (LTB_4_), a potent pro-inflammatory mediator that is closely related to neutrophil activation (54). A recent review highlighted the association between SARS-CoV-2-induced endoplasmic reticulum stress to eicosanoid pathway activation, which could augment the proinflammatory storm linked to COVID-19 (55). In this context, previous reports have shown that heparin is capable of inhibiting LBT4 signalization, thus softening the inflammatory response assigned to this molecule (56). Besides, hyperventilation-induced bronchoconstriction was attenuated through inhibition of eicosanoids production by heparin in the animal model (57). Considering COVID-19-induced multi-organ damage through hyperinflammation, the countless immunomodulatory effects of heparin beyond anti-coagulant properties improve its potential against this pandemic disease.

Another approach that is being widely discussed is the antiviral potential provided by heparins. Previous to the COVID-19 pandemic, the antiviral activity of the current drug was observed in different experimental models and viruses, such as Human Immunodeficiency Virus (HIV) and Herpes Simplex Virus (HSV) (58, 59). This antiviral effect of heparins may be related to the direct competition for binding to the cell glycoprotein receptors or through softening harmful effects caused by the infection, as already demonstrated for Zika Virus (ZIKV) (60). Growing shreds of evidence suggest that heparin also presents an antiviral effect against SARS-CoV-2. A report has shown that the cellular invasion capability of the virus can be affected in the presence of heparins (61). In this study, it was noticed that heparan sulfate (HS) derivatives, such as UFH and LMWH, at feasible concentrations for clinical application, induces conformational alteration of the SARS-CoV-2 Spike protein which is a central molecule for the host's cell invasion. Another preprint demonstrated that UFH was also capable of affecting SARS-CoV-2 entry to the host cell, impacting its infectivity parameters (62). Moreover, an *in vitro* comparative analysis found that UFH presented a higher antiviral potential against SARS-CoV-2, suggesting that it could be more beneficial than LMWH (63, 64). It was also noticed an important role of cellular HS binding to SARS-CoV-2 that promotes S protein conformational change and binding to the ACE-2 receptor, suggesting that HS could be a coreceptor for the viral invasion (65). In this context, UFH was also reported as a blocking agent of this interaction between cellular HS molecule with SARS-CoV-2, reinforcing its antiviral activity.

**Figure 1:**
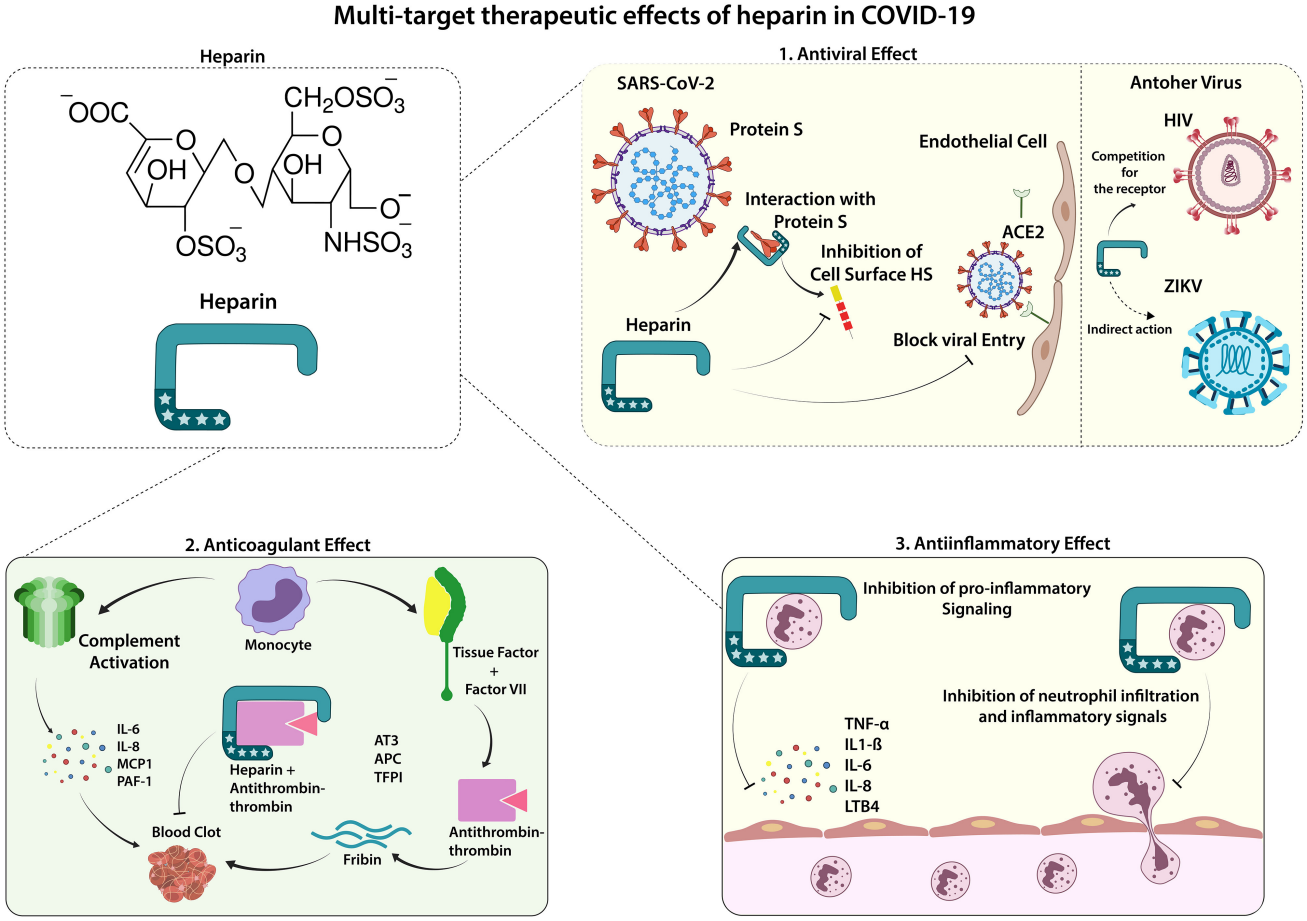
Therapeutic Effects of Heparin in COVID-19. Lung infection by SARS-CoV-2 can trigger a systemic inflammatory process, which is also associated with an increased occurrence of procoagulant factors found in severe cases of COVID-19. Low molecular weight Heparin (LMWH) shows effects in 3 main ways: 1. Antiviral effect: the entrance of SARS-CoV-2 in the endothelial and epithelial cells depends on its interaction with the cell surface heparan sulfate; thus, the binding of heparin to the viral spike protein can inhibit this interaction, decreasing viral cell invasion. Heparin has shown its antiviral effect on other viruses such as HIV (competing for the receptor) and ZIKV (indirect action, abrogating the viral-induced cytotoxic effect). 2. Anticoagulant effect: the uncontrolled blood clot formation can be controlled by the anticoagulant function inherent to heparin, mediated by the interaction of heparin and anti-thrombin-3 glycoprotein (AT3), potentiating the AT3 inactivation of thrombin, an essential factor for the formation of thrombi. 3. Anti-inflammatory effect: heparin has widely known anti-inflammatory effects, mainly canceling pro-inflammatory mediators, such as TNF-α, IL-6, and LTB4, which leads to decreased migration and activation of immune cells, preventing against the systemic inflammatory response. ACE2, Angiotensin-converting enzyme 2; APC, activated protein C; AT3, Antithrombin-III; CK, creatine kinase; CRP, C-reactive protein; ESR, erythrocyte sedimentation rate; HS, heparan sulfate; IL, interleukin; LDH, lactate dehydrogenase; PAI-1, Plasminogen activator inhibitor 1, TF, Tissue factor, TFPI, Tissue factor pathway inhibitor; TNF, Tumor necrosis factor; VII, Factor VII.

Although antiviral evidence of both LMWH and UFH against SARS-CoV-2 are still preliminary, the other protective activities against COVID-19 complications are clear. The ideal therapeutic approach for treating such complex diseases is also using a multi-target molecule that restores different SARS-CoV-2-affected pathways. The anticoagulant, anti-inflammatory, and potential antiviral effects provided by heparins increase the perspectives of using these medications for creating a better prognosis for COVID-19 affected individuals (Figure 1). Considering the findings that the antiviral effect of UFH could be stronger than LMWH, the dose of choice must be decided carefully, once UFH presents a higher risk for bleeding than LMWH. Avoiding the increased risk, the most suitable dose for both UFH and LMWH is the standard care, which can be adjusted according to the body mass index (BMI) and kidney function (66). Besides, risk factors for bleeding must be considered before prescribing the anticoagulant drug as prophylaxis. Moreover, nebulized UFH is a great possibility to improve the performance of the current drug by directing the effect to the most required site and reducing risks for unwanted impact on systemic coagulation pathways.

## Corticosteroid Dexamethasone

Based on available clinical data, around 20% of SARS-CoV-2-infected patients developed Acute Respiratory Distress Syndrome (ARDS), characterized by pulmonary pathological alterations as elevated dead space and decreased oxygenation (67–69). The massive release of proinflammatory cytokines and chemokines (5), coagulopathy and microvascular thrombosis (5, 10), hyaline membrane formation (68, 70), and intravascular DNA neutrophil extracellular traps (NETs) (71) are currently described as contributing factors for the diffuse alveolar damage commonly displayed in COVID-19 associated ARDS.

Consistently manifested by ARDS patients, pulmonary hyaline membrane formation impairs gas exchange, limits surfactant action, favors lung fibrosis, and induces lung microvascular thrombi (72, 73). Other factors that cope with thrombosis initiation and inflammation during ARDS onset are intravascular DNA neutrophil extracellular traps (NETs) and NETosis (71, 74). In addition to neutrophilia, the risk factors associated with the establishment of ARDS in COVID-19 patients were older age, organ dysfunction, and coagulopathies (75). The management of the cytokine and chemokine storm during COVID-19 represents a crucial and controversial point (76), considering that the use of systemic anti-inflammatory drugs can either inhibit the tissue damage or curb the cell-mediated immunity (77, 78).

Glucocorticoids (GCs) are steroid hormones derived from cholesterol metabolism. Both endogenous and synthetic forms of GCs share the same lipophilic chemical structure that allows the molecule to exert a broad range of endocrine effects in the organism (79). Currently, the synthetic GCs, such as dexamethasone, suffered alterations that significantly improved specificity, bioavailability, and potency, leading to a higher efficacy compared to the endogenous signaling pathway (80). The known immunosuppressive potential of dexamethasone made this anti-inflammatory molecule the first-line therapy for a great number of inflammatory diseases, such as autoimmune disorders and respiratory infections (81).

Dexamethasone mechanisms of action are characterized by exclusive binding to the classical cytosolic GC receptor (cGCR), which provides the majority of anti-inflammatory effects (82). The drug can reduce the inflammatory process by controlling the transcription of many pro-inflammatory genes that encode cytokines, cell adhesion molecules, and receptors related to inflammation (83). Dexamethasone is associated with decreased capillaries permeability, in addition to reduced neutrophil and lymphocyte migration into the inflammatory sites (84–86). An important advantage of the current drug is its extended half-life in the organism, which may decrease the time required for therapy compared to alternative GCs (74). The dose of choice is based on the desired effect, once low doses are associated with anti-inflammatory modulation and higher doses are associated with the immunosuppressive activity (87).

As previously discussed, endogenous GCs are central for metabolic homeostasis and systemic inflammatory events during tissue repair and pathogens elimination (88). Due to this fact, long-term exogenous intake of synthetic GCs can provide adverse effects, such as extreme shut down of inflammatory responses, leading to a higher susceptibility to secondary infections establishment (87, 89). Besides, GCs play a role in glucose metabolism through alterations of insulin signaling, leading ta reduced uptake and increased concentration of plasmatic glucose levels (90). This intervention in the metabolism gives rise to insulin resistance (IR) conditions caused by supra-physiologic or prolonged synthetic GCs medication, which can evolve to type-2 diabetes (T2D) and other metabolic disorders (91, 92). For this reason, synthetic GCs medication needs to be handled carefully, avoiding complications rather than therapeutic effects.

**Figure 2:**
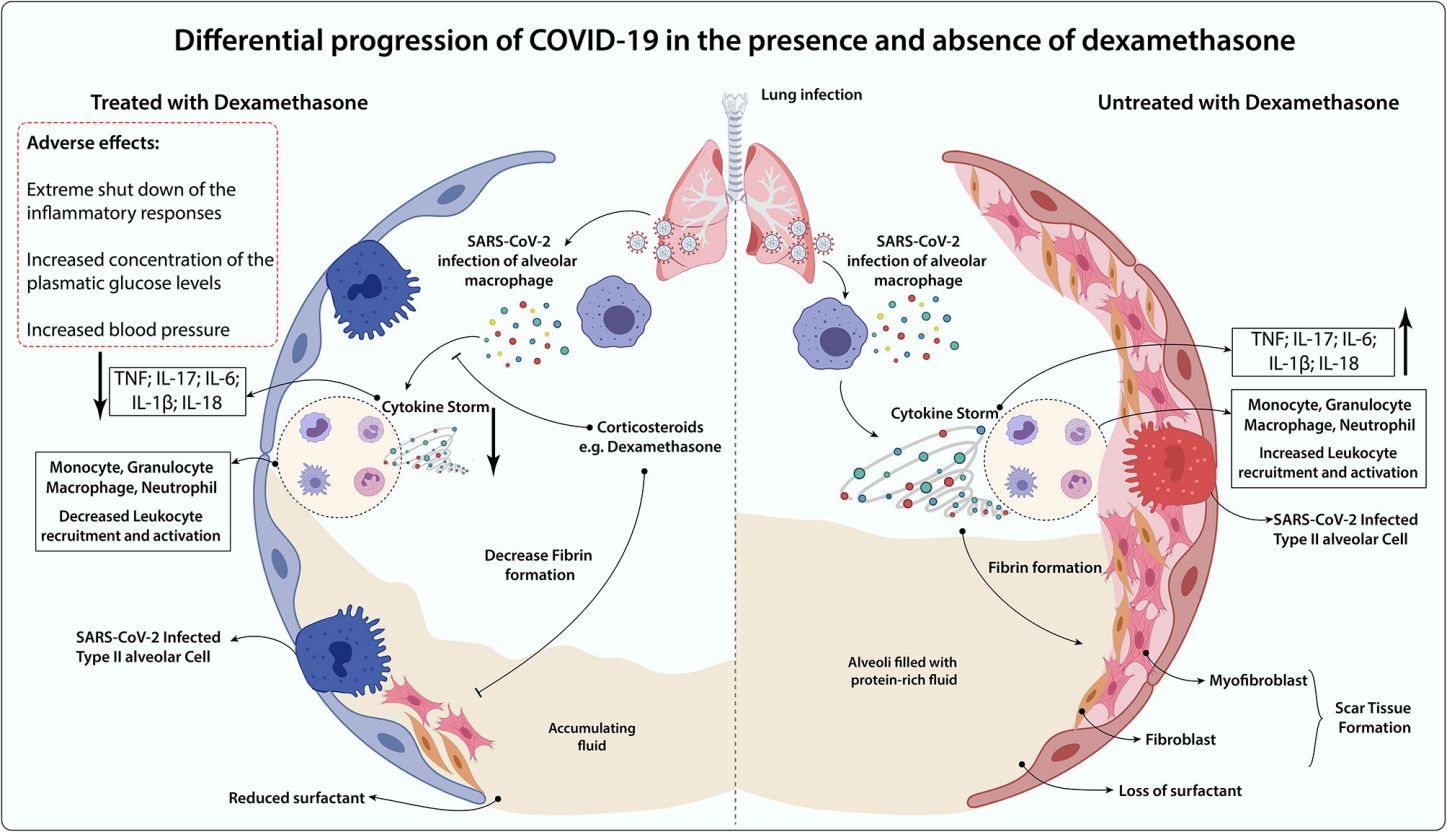
Differential progression of COVID-19 in the presence and absence of dexamethasone. In the absence of an anti-inflammatory treatment, COVID-19-induced lung dysfunction is triggered by hyperactivation of alveolar macrophages infected by SARS-CoV-2 and immune cells (Monocytes, Granulocytes, Macrophages, Neutrophils) recruitment to the lung surroundings, which leads to the massive secretion of inflammatory mediators (TNF, IL-17, IL-6, IL-1b, IL-18) in the cytokine storm release. The persistence of the inflammatory process gives rise to the increased fibroblasts and myofibroblast invasion throughout the scar tissue formation, loss of natural pulmonary surfactant, and increased alveolar fluid. Dexamethasone administration may benefit through the impairment of cytokine storm occurrence and leukocyte lung infiltration, which decreases tissue fibrosis and alveolar fluid accumulation. However, this glucocorticoid is associated with immunosuppression, augmented blood pressure, and glycemia as major side effects. Thereby, it may be detrimental for some groups, including non-severe COVID-19 cases, diabetics, and hypertensive subjects.

At first, it was not clear whether patients diagnosed with COVID-19 could take advantage of the use of dexamethasone, especially taking into account the above-mentioned points (93). Reports have shown that dexamethasone medication against influenza-induced pneumonia, SARS-CoV-1, and Middle East respiratory syndrome coronavirus (MERS-CoV) presented harmful effects that could negatively impact the disease prognosis (94–97). However, specifically in patients who develop ARDS during COVID-19, dexamethasone treatment showed to be effective in down-regulating systemic and pulmonary inflammation, restoring tissue homeostasis by accelerating resolution of diffuse alveolar damage, leading to protection against extrapulmonary organ dysfunction (98, 99). Besides, experimental and clinical studies have demonstrated that GR-α expression in myeloid cells of bronchoalveolar lavage is associated with significant protection against severe COVID-19 symptoms, especially through reduction of NETosis and lung neutrophilic inflammation (100). Of note, when lung damage has already occurred, the case fatality rate of COVID-19 is high (101). If dexamethasone could alleviate the clinical progression at this stage, then the therapy may decrease the cases of severe illness and therefore lower the case fatality rate of COVID-19 (102). A comparative scenario of COVID-19 progression in the presence and absence of dexamethasone was summarized (Figure 2).

Faced with the complex scenario of severely ill patients with COVID-19, diverse protocols employing complementary treatments (94) have been developed, some of them using GCs for the treatment of hospitalized patients with COVID-19 (103). Members of the WHO and the Chinese Thoracic Society have conflicting opinions regarding the use of corticosteroids in COVID-19 (104). According to the WHO guidelines, dexamethasone should only be used under clinical trial conditions (105). Russell and colleagues concluded that there is no reason to expect that individuals with COVID-19 will benefit from corticosteroid treatment, based on the increased mortality and risk of secondary infection in influenza, impaired clearance of SARS-CoV, and MERS-CoV (93). However, the high potential of dexamethasone for cytokine storm mitigation brought proposals to overcome the impairment of viral clearance and increased risk of developing secondary infections, and the main in-progress clinical trials are summarized in Table 2.

**Table 2:** Studies on dexamethasone for COVID-19.

**Author, year, study design, country**	**Number of patients**	**Treatment**	**Patient characteristics**	**Results**
Selvaraj et al., Cases series; USA	23	Dexamethasone: 4 mg	Age: 60 y; Comorbidities: 38.09% hypertension; 61.9% diabetes; BMI: 28.68 kg/m^2^	CS prevented the progression of hypoxic respiratory failure in moderate to severely ill patients
Recovery Group 2020; Multicentric; Controlled, open-label trial; UK	6,425	Dexamethasone (6 mg once daily - 10 days)	Age: 66.1 y; Comorbidities: 24% diabetes; 27% CDV; 21% chronic lung 56% having at coexisting illness	In the dexamethasone group, the incidence of death was lower than that in the usual care group among pts receiving IMV
Tomazini et al., 2020; Multicentric, randomized, open-label, clinical trial; Brazil	299	Dexamethasone (10 mg – 5 days)	Age: 61 y: Comorbidities: 60.3% hypertension; 37.8% diabetes; 30.5% obesity	The use of standard care compared with standard care alone resulted in a significant increase in the number of ventilator-free days over 28 days.

*AKI, acute kidney injury; ARDS, acute respiratory distress syndrome; CDV, cardiovascular diseases; CS, corticosteroids; ICU, intensive care unit; IMV, invasive mechanic ventilation; MTP, methylprednisolone; NIV, non-invasive ventilation; Pts, patients; UK, United Kingdom; USA, United States America; y, years*.

The preliminary results of a large randomized, controlled, open-label trial conducted in the United Kingdom are in favor of dexamethasone use. In this trial, Dexamethasone arm constituted 2,104 patients receiving 6 mg dexamethasone (oral or intravenous) once daily for up to 10 days and 4,321 patients receiving standard care. Dexamethasone reduced mortality by 35% in patients receiving invasive mechanical ventilation. Besides, the prospective meta-analysis from the WHO Rapid Evidence Appraisal for COVID-19 Therapies (REACT) Working Group recommended the independent use of corticosteroids in patients with COVID-19. An important correlation between the administration of systemic GCs and reduced mortality was found among critically ill patients with COVID-19 (106). Selvaraj et al. reported that short-term use of dexamethasone by hospitalized patients with COVID-19 was well tolerated and increased the patients' prognosis (107).

Although dexamethasone promising performance for severe COVID-19, there are still concerns regarding this anti-inflammatory drug prescription for indiscriminately cases. Until now, the available data suggests a beneficial role of dexamethasone treatment on hospitalized patients, especially for those who received intensive oxygen therapy (108, 109). A possible explanation can be based on the association between hyperinflammation and the development of pulmonary damage, increasing urgency for ICU. The excessive inflammation needs to be handled by a strong anti-inflammatory treatment, which could be accessed by dexamethasone therapy. In the absence of an unbalanced inflammatory process as in the early stage of the disease, dexamethasone treatment might disturb the development of the host's natural immunity and abrogate anti-viral response, which could lead to a delayed viral clearance.

Besides the previously discussed impairment of viral clearance when applied in the early stage of the disease (95), the increasing risk of secondary infections associated with high doses of systemic GCs was noticed (87, 89). A report regarding *Strongyloides* hyperinfection, a neglected nematode disease, brought the current concern about dexamethasone application without concomitant vermifuge use (110).

Besides that, GCs modulation of glucose metabolism leads to intensive care on diabetic patients during the treatment (92). As discussed worldwide, diabetes is a major risk factor for severe COVID-19 and dexamethasone treatment in these individuals could be associated with the development of hyperglycemic condition (111–113). The increased concentration of glucose levels in the bloodstream could be associated with a poor prognosis of the disease, considering the recent discovery about SARS-CoV-2 dependency of glucose for viral replication *in vitro* (114). With increasing glucose concentration, the viral load increased concomitantly, favoring the infection and the severity of the disease (114). Besides, hypertensive patients, who are also at risk for COVID-19 severity, should be at constant monitoring during dexamethasone treatment, once GCs-induced hypertension is frequently observed (115). High dexamethasone doses are associated with increased sodium retention, which leads to the elevation of blood pressure, in addition to chemical alteration of peripheral nerves homeostasis (87, 116, 117). This effect could lead to complications rather than COVID-19 treatment, increasing the associated mortality risks of the affected individuals.

## Conclusion

Severe cases of COVID-19 are marked by intense inflammation and the presence of thrombotic events. Exacerbated inflammation that mediates the characteristic cytokine storm observed in the severe COVID-19, and the blood clots in the lungs that can compromise oxygenation, lead to worsening clinical outcomes of SARS-CoV-2 infection. Several recent reports have demonstrated a beneficial effect of the use of heparin/low molecular weight heparin and corticosteroids, such as dexamethasone, on mortality in COVID-19.

The advantageous and successful effect of heparin underlying treatment of COVID-19 patients could be explained not only by its anticoagulant properties but also due to its non-anticoagulant mechanisms, which include anti-viral and anti-inflammatory actions such as (I) decrease of SARS-CoV-2 host cell entry, (II) inhibition of pro-inflammatory cytokines and chemokines, (III) inhibition of vascular permeability and leukocyte migration.

Despite the controversial role of corticosteroids in treating severe infectious diseases, several clinical studies have provided increasing evidence that dexamethasone could function widely as an available treatment for the most severely ill patients with COVID-19. However, many clinically important questions remain open, and determination of optimal initiation period, dosing, and duration of the dexamethasone treatment might be considered to avoid serious adverse effects during COVID-19 management.

While UFH or LMWH are indicated as prophylactic agents for the initial phase of the disease, which could impair clots formation in addition to abrogate viral cell entry, dexamethasone must be prescribed only for severe cases, when the disease reaches a highly inflammatory state. Taking this into account, we suggest that the multitarget impact of heparin as an anti-viral, antithrombotic and anti-inflammatory drug in the early stage of the COVID-19 could significantly reduce the need for dexamethasone treatment in the initial phase of this disease. If the standard treatment of heparins fails on protecting against severe illness, dexamethasone must be applied as a potent anti-inflammatory shutting-down the uncontrolled and exacerbated inflammation.

Overall, the association of anti-coagulant heparin and the corticosteroid dexamethasone could be a very effective and promising therapeutic tool in avoiding COVID-19 complications when used for severely ill patients.

## Author Contributions

GP-d-N, HB-d-M, SF, GK, and KM wrote different sections of the manuscript. KM revised, wrote, and prepared the manuscript. IS prepared the figures. All authors listed have made a substantial, direct and intellectual contribution to the work, and approved it for publication.

## Conflict of Interest

The authors declare that the research was conducted in the absence of any commercial or financial relationships that could be construed as a potential conflict of interest.
